# Sub-Mucus Window Resection of the Cartilagious Septum

**Published:** 1907-11

**Authors:** R. H. T. Mann

**Affiliations:** Texarkana, Ark-Tex.


### SUB-MUCUS WINDOW RESECTION OF THE CARTILAGINOUS SEPTUM.*

By R. H. T. MANN, M.D., Texarkana, Ark.-Tex.

Until the Sub-Mucus Window Resection operation for marked deflections of the nasal septum was devised there was no surgical procedure which gave relief for these conditions in nearly as large a per cent. of cases, and with as little inconvenience to the patient. The Asch operation gave relief in from 50 to 60 per cent. of cases of deflections occurring in the cartilag-

(*Author's Abstract of paper read before section on Eye, Ear, Nose and Throat of the Medical Association of the South-East, Hot Springs, Ark., Oct. 9th.)

inous septum, but it presented the distinct disadvantage of having to be performed wholly in the dark after the first incision was made, as the nasal cavity was filled with blood. Another very great disadvantage was the wearing of a nasal splint for about six weeks.

Many deflections of the nasal septum are of slight degree and cause no inconvenience. These should be left alone, especially is this true in large noses.

The operation is contra-indicated in patients in the advanced stages of tuberculosis and the primary or secondary stages of syphilis, and also patients suffering from any acute suppurative condition of the nose. Also contra-indicated in old people who have become accustomed to faulty nasal breathing and children under twelve years of age. It is not yet known what effect the removal of cartilaginous septum will have on the development of the noses of children.

The operation is indicated in all conditions of the nose where the deflections cause any annoyance, such as mouth-breathing, headache, sneezing, a constant secretion, asthmatic attacks due to nasal stenosis. A better knowledge of the parts has revealed the fact that most of the so-called spurs are slight deflections.

The preparations for the operation should be as thorough as for major operations, as infection, no doubt, produces a severe complication. The operation presents many difficulties at first to the beginner. In fact, it is not in any sense an easy operation. After the technique, however, has been mastered it becomes much easier. The operation can be performed with cocaine anaesthesia with little pain if one waits sufficient time for the cocaine to take effect and is careful about cocainizing the parts. Cocaine anaesthesia is best produced by weak injections of the drug beneath the mucus membrane and the perichondrum on each side. This also greatly facilitates the elevation of the perichondrium from the cartilage. A very weak solution of adrenalin may be added to the cocaine solution and injected at the same time to prevent hemorrhage.

In nervous patients it is no doubt better to use 1-4 of morphia and 1-50 of atropine sulphate hypodermically one-half hour before the operation. Should pain occur during the operation a four per cent. solution of cocaine can be applied on a cotton swab directly to the parts. The recumbent position is best, as the patient is not likely to complain of faintness which often occurs at the beginning of the operation in the sitting posture.

The Kirstein, or some form of light, worn on the head is better. Any light, however, which will illuminate the deep parts of the nose will answer.

The first step of the operation is an incision in the mucus membrane from the floor of the nose to within 1-8 of an inch of the bridge. This incision should be just far enough back from the beginning of the cartilaginous septum to allow ample cartilage to remain for the support of the tip of the nose. However, in deflections which extend to the tip, the cartilage can all be removed with little sagging of the nose, not enough to cause deformity. This first incision should extend through the perichondrium and into the cartilage. The perichondrium is next elevated from the cartilage at first with a sharp elevator, then a blunt one. The incision is next made through the cartilage but not through the perichondrium nor mucous membrane on the other side. The perichondrium is elevated from the cartilage on this side as well, after which the cartilage can be easily removed with a Ballenger's swivel knife, the mucus membrane being held back on each side with a Killian's speculum.

In elevating the perichondrium around sharp curves, it is better to remove the cartilage up to the curve when the cartilage can be pushed aside and the separation be made very easily, greatly lessening the danger of a perforation. In bony deflections the bone can be removed with a chisel and bone forceps. No part of the crook should be left whether it be cartilage or bone.

The greatest danger encountered in the performance of the operation is a perforation in the initial incision. Some perforations are to be expected in the beginning, but these become less as the technique is mastered. The operation when correctly performed gives uniformly good results.

The after treatment consists in packing the nose with gauze strips which have been dusted with bismuth sub-nitrate. These can be removed from the side on which the incision was not made within 24 hours and from the other side in seventy-two hours.

Should granulating tissue spring from the point of the incision 40 grs. of nitrate of silver to an ounce can be applied to this for a few days. A little absorbent cotton can be placed in the end of the nose to prevent dust entering until the wound has entirely healed.

Patients can leave the hospital by the end of the third day and resume their usual avocations at the end of a week.

